# Serum levels of bone turnover markers including calculation of *Z*-scores: Data from a Dutch healthy reference cohort

**DOI:** 10.1016/j.bonr.2023.101724

**Published:** 2023-11-12

**Authors:** Mark Siderius, Suzanne Arends, Anneke Muller Kobold, Lucie Wagenmakers, Karin Koerts, Anneke Spoorenberg, Eveline van der Veer

**Affiliations:** a Rheumatology and Clinical Immunology, University of Groningen, University Medical Center Groningen, P.O. Box 30.001, 9700 RB Groningen, the Netherlands; b Laboratory Medicine, University of Groningen, University Medical Center Groningen, P.O. Box 30.001, 9700 RB Groningen, the Netherlands

**Keywords:** Bone turnover markers, Bone metabolism, Z-scores, Reference values, Treatment monitoring

## Abstract

**Introduction:**

Bone turnover markers (BTM) are biochemical compounds reflecting different stages of bone metabolism. Their levels change with age and differ between males and females. This makes clinical interpretation and comparison more difficult. Therefore, our aim was to establish BTM reference values which can be used to calculate *Z*-scores for use in daily clinical practice.

**Methods:**

Serum markers of collagen resorption, bone formation/regulation, collagen formation and bone mineralization (sCTX, OC, PINP and BALP, respectively) were measured in non-fasting volunteers without bone-related abnormalities. Raw data was plotted and gender-specific age cohorts were established with their respective means and standard deviations (SD). *Z*-scores can be calculated using these reference values to correct for the influence of age and gender on BTM.

**Results:**

In total, 856 individuals were included of which 486 (57 %) were female. Individuals were aged between 7 and 70 years. Highest serum levels of BTM were found in childhood and puberty. Peak levels are higher in boys than girls and prevail at later ages. In adults, BTM levels decrease before reaching stable nadir levels. In adults, 10-year reference cohorts with means and SD were provided to calculate *Z*-scores.

**Conclusion:**

With our data, *Z*-scores of sCTX, OC, PINP and BALP can be calculated using reference categories (for age and gender) of Caucasian healthy volunteers. Clinicians can use BTM *Z*-scores to determine whether there are changes in bone turnover physiology beyond those expected during aging. BTM Z-scores facilitate harmonization of data interpretation in daily clinical practice and research.

## Highlights

• Bone turnover markers (BTM) are biochemical compounds reflecting different stages of bone metabolism.
• Absolute BTM levels change with age and differ between males and females complicating data interpretation and comparison.
• BTM *Z*-scores facilitate harmonization of data interpretation in daily clinical practice and research.

## 1 Introduction

Bone turnover markers (BTM) are biochemical compounds that in vivo mirror the core processes of bone metabolism. BTM themselves can be enzymes involved in either of the process, proteins or degradative products. Their dynamic character provides additional information with regard to the current status and balance of bone metabolism (Greenblatt et al., 2017). This in contrast to the data obtained via dual-energy X-ray absorptiometry (DEXA) on bone mineral density (BMD), which mirrors the bone status or outcome.

The intrinsic reflective character of BTM, however, complicates its interpretation. BTM levels are not only different between females and males, but are also known to change with age influenced by several physiological processes such as growth and changes in hormone secretion (Eastell, 2005; Lephart, 2018). Measurement of BTM is clinically relevant and common in the fields of rheumatology, nephrology and oncology where they attribute both during the diagnostic workup as well as during treatment monitoring (Greenblatt et al., 2017; Bhattoa, 2018). BTM are, however, most known for their importance in monitoring osteoporosis treatment. Some studies indicated that BTM may predict fracture risk independently from BMD (Vasikaran et al., 2011; van der Veer et al., 2014).

Reference values are essential for interpreting BTM and its need was already discussed in 2011 (van der Veer et al., 2014). So far, several studies have been published about the establishment of reference values. Most studies described reference values in very specific populations of individuals e.g. young healthy woman, children or older men (Eastell et al., 2012; Yang and Grey, 2006; Alberti et al., 2011; Glover et al., 2008; Chubb et al., 2015) or described the course and absolute values for one specific BTM (Yoo et al., 2018; Avbersek-Luznik et al., 2007; Hannemann et al., 2013; Bayer, 2014). Based on the study of health in Pomerania, reference intervals were described for commonly used BTM, i.e. collagen resorption marker serum C-telopeptide cross-link of type I collagen (sCTX), collagen formation marker procollagen type I N-terminal peptide (PINP) and regulator of mineralization marker bone-specific alkaline phosphatase (BALP), as normative data to interpret bone turnover in adult men and pre- and postmenopausal women. However, they did not provide means and standard deviations (SD) of reference cohorts necessary to calculate *Z*-scores (Michelsen et al., 2013).

In addition, some studies calculated T-scores for BTM using a fixed peak value as reference (Gossiel et al., 2020; Gossiel et al., 2018). This concept is widely known for interpreting BMD data and the definition of osteoporosis using BMD T-scores (National Osteoporosis Foundation, 2013). However, the official position from the International Society for Clinical Densitometry states to use *Z*-scores rather than T-scores in males before the age of 50 and in females prior to menopause for interpreting BMD data (Shuhart et al., 2019). *Z*-scores are defined as the number of SD from the mean of a reference population matched for age and gender. Using *Z*-scores, it is possible to interpret and directly compare individual values. In cross-sectional studies, the use of *Z*-scores allows for combined statistical analysis of individuals of different ages and gender, to estimate effects at group level (Arends et al., 2011). In long-term longitudinal studies, *Z*-scores make it possible to evaluate the long-term effect over time excluding the natural course effect of age (Arends et al., 2012). An additional clinically useful advantage of Z-scores is the ability to quantify changes in both bone turnover physiology beyond those expected during growth or aging.

Thus far, a study providing reference values for BTM of the different processes of bone metabolism and the possibility to calculate *Z*-scores resembling daily clinical practice is still missing. Therefore, the aim of this study was to describe the establishment of reference values for BTM which reflect the process of bone metabolism, i.e. resorption, formation/regulation and mineralization. Additionally, to provide mean and SD values for the calculation of *Z*-scores in a Dutch population based on widely used BTM assays and/or automated immunoassay platforms which can be used in daily clinical practice. Finally, we provided examples on the use and interpretation of BTM *Z*-score, exempli gratia (e.g.) when monitoring medication persistence of anti-osteoporotic treatment.

## 2 Methods

### 2.1 Samples for BTM from reference population

From 2006 to 2015, healthy volunteers, both children and adults, were recruited. Children were recruited via informative meetings at schools. Adults were recruited from control groups from BTM studies and amongst laboratory personnel. All volunteers were Caucasian. Inclusion of volunteers was evaluated using a questionnaire with health and bone-related questions and screened by an expert in the field of bone metabolism. Excluded were individuals with self-reported fractures, pregnancy and lactation, malnutrition, immobility, chronic diseases including liver disease, renal impairment, thyroid disorders diabetes mellitus, bone-related diseases or medication use affecting the bone metabolism (e.g bisphosphonates, glucocorticoids, anticonvulsants, coumarin derivatives, hormone replacement therapy). Furthermore, 25OHvitD was above 50 nmol/L and osteoporosis (BMD T-score > −2.5) was checked and excluded after 50 years of age.

Blood samples were drawn at variable time points to take into account circadian variation using a vacutainer tube without additives. Samples were kept on ice and transported to the laboratory and within one hour, they were centrifugated, aliquoted and stored at −80 °C until analysis. Analysis was performed within one week. Additionally, samples were checked for serum 25 hydroxy cholecalciferol (vitamin D) levels >50 nmol/L.

All volunteers, and in the case of children also their legal representative, provided written informed consent according to the Declaration of Helsinki. The Medical Ethics Review Board (University Medical Center Groningen (UMCG)) concluded that this study was not considered as clinical research with human subjects, and waived the need for ethical approvement or consent.

### 2.2 Analyses of BTM during inclusion of reference cohort (2006–2015)

The following BTM were measured in serum; collagen resorption marker sCTX, bone formation/regulation marker osteocalcin (OC), collagen formation marker PINP and regulator of bone mineralization BALP.

From 2004 to 2021, sCTX was analyzed using the β-crossLaps immunoassay on the Elecsys immunoanalyzer. This assay had an inter-assay coefficient of variation of 2.7–7.6 %.

From 2006 to 2020, intact osteocalcin was analyzed using a Radio Immuno Assay from Diasource (DIAsource ImmunoAssays SA, Louvain-la-Neuve – Belgium). This assay had an inter-assay coefficient of variation of 9.4 %.

Total PINP was analyzed using the PINP Orion radioimmunoassay (Orion Diagnostica, Espoo, Finland). This assay had an inter-assay coefficient of variation of 9.0 %.

Since 2003, BALP was analyzed using the enzyme-linked immunosorbent assay (Metra Biosystems, Mountain View, CA, USA, New owner Quidel MicroVue, Athens, USA)). This assay had an inter-assay coefficient of variation of 5.5 %. This assay uses a standard containing BALP purified from osteosarcoma SAOS-2 cells.

In addition to the BTM, 25 hydroxy cholecalciferol (vitamin D) was measured in all individuals, by extraction followed by radioimmunoassay (DiaSorin, Stillwater, MN, USA).

### 2.3 BTM analyses after inclusion of reference cohort

In 2021, the laboratory switched to another immunoassay platform for the measurement of sCTX, namely the Cobas e601 (Roche Diagnostics GmbH, Mannheim, Germany). After validation and method comparison (EP9 and modified EP 5 A, Simple precision), Passing and Bablok analysis revealed the following relation: Cobas = 0.99× Elecsys −8.11 (ng/mL). The Cobas assay has a inter assay variation of 2.7–7.6 % over the measuring range. This method has been standardized against reference standards precisely defined by weighing out synthetic peptide. The relation (with its 95 % confidence intervals including 1 and 0 for slope and intercept respectively) between the old and the new sCTX assay demonstrated that previously measured values from the reference cohort could be used without being recalculated.

In 2020, the laboratory switched to the Elecsys N-MID Osteocalcin assay on the Cobas e601 (Roche Diagnostics GmbH, Mannheim, Germany) After validation and method comparison (EP9 and modified EP 5 A, Simple precision), Passing and Bablok analysis revealed the following relation: Cobas = 1.29× DiaSource +1.61 (ng/mL). The Cobas assay has a inter assay variation of 0.85–1.26 % over the measuring range. This assay has been standardized, according to the Roche kit insert information, against an in-house reference standard: osteocalcin in analyte-free human serum matrix. Since the new assay resulted in 29 % higher values, we used this factor to recalculate the previously measured values from the reference cohort.

In 2021, the laboratory switched to another assay, the Elecsys total PINP assay on the Cobas e601 (Roche Diagnostics GmbH, Mannheim, Germany) After validation and method comparison (EP9 and modified EP 5A, Simple precision), Passing and Bablok analysis revealed the following relation: Cobas = 1.05x Orion RIA-3.98 (ng/mL). This relation (with its 95 % confidence intervals including 1 and 0 for slope and intercept respectively) demonstrated that previously measured values from the reference cohort could be used without being recalculated. This relation was demonstrated that previously measured values from the reference cohort could be used without being recalculated. The Cobas assay has a inter assay variation of 1.63–2.16 % over the measuring range. This method has been standardized against reference standards precisely defined by weighing native PINP into an analyte-free human serum matrix.

Assays for assessment of serum levels of BALP have remained unchanged.

BTM were measured in a NEN-EN-ISO 9001:2015 certified and NEN-EN-ISO 15189:2012 accredited laboratory.

### 2.4 Statistical analysis

Passing and Bablok analyses were performed using Excel and the Analyse-it add on software. Descriptive statistics (mean and SD) were used to express the overall distribution of BTM in the population across age and gender. Based on this, age-specific cohorts were established separately for males and females, and subsequently their respective means and SD were calculated for adults. *Z*-scores can be calculated as (BTM value of individual patient – mean BTM value of the of reference group matched for age and gender) / SD of matched reference cohort. For children, we decided to only plot the data to show the natural course of BTM in boys and girls, since we had no information available on the Tanner stage to account for puberty status and some year-cohorts were too small to provide accurate means and SD for the calculation of the reference values.

## 3 Results

### 3.1 BTM reference population

**Fig. 1:**
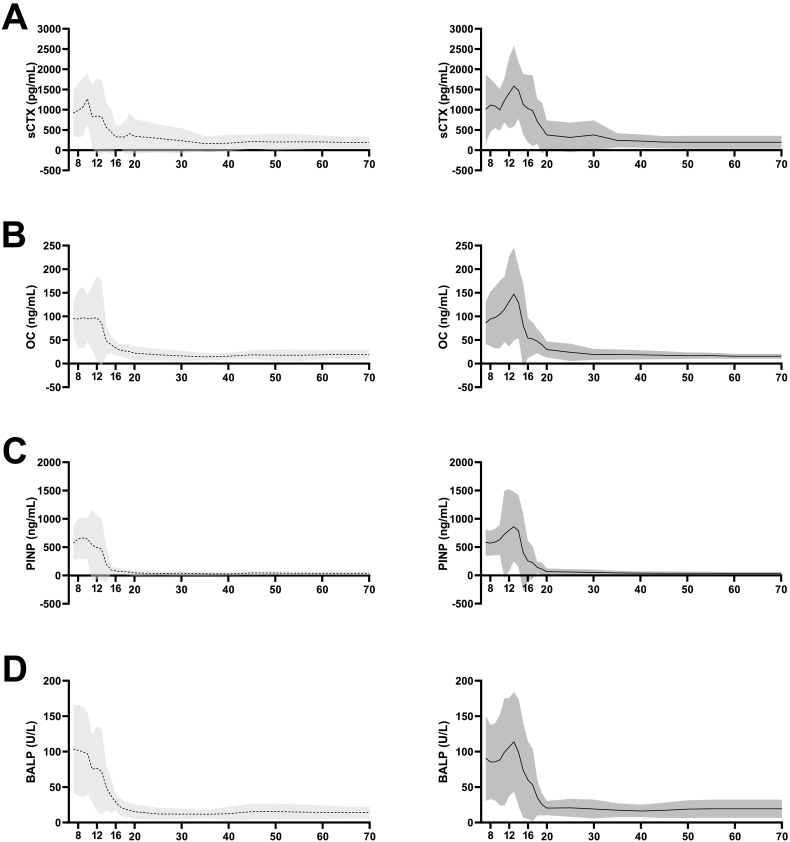
Course at group level (presented as mean and ± 2SD) of serum levels of sCTX (A), OC (B), PINP (C) and BALP (D) in females and males of a healthy reference cohort (−--; female, —; male). Figure represents the overall course of BTM, however, should not be interpreted as reference curves.

In total, 856 individuals were included of which 486 (57 %) were female. Individuals were aged between 7 and 70 years. Females as well as males showed large variability during aging, but all four markers had comparable patterns (Fig. 1).

### 3.2 BTM in childhood and puberty (7–20 years of age)

As expected, serum levels of BTM in non-adults (<20 years) showed large variability, therefore year-specific mean and SD were warranted and established for this group.

Collagen resorption marker sCTX levels showed a peak level in girls and boys. Girls showed peak levels at the age of 10–11 years. The average level during this year was 1269.4 ± 310.5 pg/mL. Peak levels in boys were seen around the age of 13–14, with average peak levels of 1582.6 ± 500.1 pg/mL (Fig. 1A).

Bone formation/regulation marker OC average levels did not show a peak level in girls, but rather a plateau phase till the age of 13 years. This level during these years was 96.1 ± 39.9 ng/mL. Boys did show an increasing trend of OC and reached peak levels of 147.5 ± 49.2 ng/mL at the age of 13–14 years. OC levels were similar for both genders between the ages of 7–11 years (Fig. 1B).

The average level of collagen formation marker PINP had a course similar to OC. Girls did not show a peak level but rather a plateau phase till the age of 12 years. The average level during these years was 623.6 ± 25.3 ng/mL. Boys did show an increasing trend of PINP and reached peak levels of 859.4 ± 306.5 ng/mL at the age of 13–14 years. Collagen formation was very similar for both gender between the ages of 7–11 years. After peak levels, PINP decreased quickly (Fig. 1C).

At younger ages, mineralization reflected by regulator BALP was relatively higher in girls than boys. In girls, BALP decreased gradually in a step wise manner after the age of 10 years. Average peak levels of 103.2 ± 30.5 were already present at or before the age of 7. In boys, BALP increased with age and showed peak levels of 113.9 ± 35.1 U/L between the ages of 13–14 years. After reaching peak levels, BALP decreased gradually in a step wise manner (Fig. 1D).

### 3.3 BTM in adults (20–70 years of age)

Adults (>20 year) had less variability of serum BTM and therefore could be grouped in 10-year intervals, with exception of OC which was grouped in 20-year intervals for males.

In females, sCTX levels tended to increase minorly between the ages of 20–25 before decreasing, reaching stable nadir levels of sCTX between the ages of 35–45, with average levels of 161.1 ± 89.5 ng/mL. In males, sCTX levels stepwise decreased with age, stable nadir levels were not reached before 70 years of age (Fig. 1A, Table 1, Table 2).

**Table 1:** Reference values of serum BTM levels in females by ascending age in 10-year cohorts. The preferred reference cohort is the one in which the age of the individual is most equal to the mean age of the reference cohort.

sCTX (pg/mL)			OC (ng/mL)			PINP (ng/mL)			BALP (U/L)		
Age	N	Mean ± SD	Age	N	Mean ± SD	Age	N	Mean ± SD	Age	N	Mean ± SD
19–25	18	440.0 ± 234.0				20–25	40	49.1 ± 27.2	20–25	22	16.7 ± 5.3
20–30	18	345.0 ± 212.8	20–30	46	22.0 ± 7.6	20–30	54	45.0 ± 25.5	20–30	33	15.2 ± 5.1
25–35	16	290.5 ± 173.8	25–35	19	18.7 ± 5.9	25–35	20	33.8 ± 15.6	25–35	17	12.1 ± 4.3
30–40	15	233.3 ± 155.3	30–40	19	16.4 ± 5.4	30–40	16	37.6 ± 20.4	30–40	23	11.9 ± 3.8
35–45	22	161.1 ± 89.5	35–45	26	14.4 ± 3.9	35–45	24	31.2 ± 18.5	35–45	30	11.8 ± 2.8
40–50	14	173.2 ± 101.6	40–50	20	15.5 ± 3.6	40–50	18	27.7 ± 13.0	40–50	21	12.5 ± 4.9
45–55	6	213.0 ± 86.0	45–55	13	18.7 ± 4.7	45–55	12	45.1 ± 17.9	45–55	15	15.4 ± 5.7
50–60	32	200.6 ± 102.5	50–60	48	17.8 ± 6.0	50–60	48	42.5 ± 15.8	50–60	50	15.7 ± 5.4
55–65	60	204.5 ± 94.2	55–65	79	17.6 ± 6.0	55–65	80	39.5 ± 15.1	55–65	82	14.7 ± 5.4
60–70	53	204.6 ± 79.3	60–70	64	18.6 ± 4.9	60–70	65	38.6 ± 14.7	60–70	64	14.2 ± 4.9

**Table 2:** Reference values of serum BTM levels in males by ascending age in 10-year cohorts. The preferred reference cohort is the one in which the age of the individual is most equal to the mean age of the reference cohort.

sCTX (pg/mL)			OC (ng/mL)			PINP (ng/mL)			BALP (U/L)		
Age	N	Mean ± SD	Age	N	Mean ± SD	Age	N	Mean ± SD	Age	N	Mean ± SD
20–25	13	384.7 ± 155.0				20–25	10	77.8 ± 28.9	20–25	13	19.3 ± 4.1
20–30	21	375.4 ± 180.1	20–30	22	29.8 ± 8.5	20–30	24	66.3 ± 27.9	20–30	28	20.4 ± 5.0
25–40	15	314.0 ± 183.6	25–35	12	23.9 ± 9.1	25–35	21	58.8 ± 25.7	25–35	24	20.8 ± 6.3
30–50	23	247.1 ± 90.5	30–50	31	19.5 ± 5.8	30–40	19	51.4 ± 24.8	30–40	20	19.0 ± 6.6
35–55	27	241.9 ± 88.0	35–55	36	19.3 ± 5.5	35–45	24	42.0 ± 18.0	35–45	25	17.5 ± 4.9
40–60	31	225.5 ± 81.5	40–60	40	18.5 ± 4.9	40–50	24	37.6 ± 15.9	40–50	32	16.3 ± 4.4
45–65	27	197.6 ± 74.7	45–65	34	17.7 ± 4.2	45–55	32	37.5 ± 15.3	45–55	38	17.2 ± 5.5
50–70	19	194.3 ± 79.0	50–70	22	16.7 ± 3.4	50–60	42	36.9 ± 12.9	50–60	41	18.9 ± 6.3
						55–65	36	36.1 ± 12.4	55–65	27	19.6 ± 6.5
						60–70	21	35.2 ± 11.0	55–70	21	19.4 ± 6.4

For females, stable nadir levels of OC were reached between the ages of 35–45, with average levels of 14.4 ± 3.9 ng/mL. Thereafter, levels tended to increase minorly before remaining stable. For males, OC stable nadir levels were observed between the ages of 60–70 years, with average levels of 15.5 ± 2.5 ng/mL (Fig. 1B, Table 1, Table 2).

For females, stable nadir levels of PINP were reached between the ages of 40–50, with average levels of 27.7 ± 13.0 ng/mL. Just as observed in OC, thereafter, levels tended to increase minorly with age before remaining stable. For males, OC stable nadir levels were observed between the ages of 60–70 years, with average levels of 35.2 ± 11.0 ng/mL (Fig. 1C, Table 1, Table 2).

For BALP, stable nadir levels were reached between the ages of 35–45 for females or 40–50 for males, with average levels of 11.8 ± 2.8 and 16.3 ± 4.4 U/L, respectively (Fig. 1D, Table 1, Table 2).

Although, in general area of mean and +/− 1 SD levels of BTM showed great overlap after the age of 30 for both females and males, absolute levels and course were still variable. Especially in males, OC levels were most homogenous with aging.

### 3.4 Age- and gender-specific BTM reference values and *Z*-scores

Mean and SD of BTM levels were calculated for different age groups in both females as males. BTM Z-scores can be calculated using the established equation explained in the statistical paragraph and accompanying reference tables (shown in Table 1). For the adult population, gender-specific reference were established in 10- or 20-year interval cohorts. Overlapping of interval cohort increases and allows for a more subtle change between age groups. The preferred reference cohort is the one in which the age of the individual is most equal to the mean age of the reference cohort. For example, the preferred reference cohort for a male of 30 years of age is the reference cohort for the ages 25–35 not 20–30 or 30–40.

### 3.5 Examples

Fluctuations in absolute BTM values during growth and the importance of *Z*-scores in the interpretation of BTM data can be illustrated using the following example. A 9 year old girl with a serum BALP level of 99.0 U/L has a Z-score of −0.03. At the age of 19, her BALP level is 17.3 U/L, which also corresponds to a Z-score of −0.03. Therefore, despite a large decrease in absolute level of BALP, her *Z*-score has not changed when taking into the account the normal effect of growth on BTM. As another example, a male of 40 years with a serum PINP level of 70.0 ng/mL has a *Z*-score of 1.56. At the age of 60, his PINP levels remained 70.0 ng/mL, which corresponds to a Z-score of 2.73. Despite the absence of change in absolute levels of PINP, Z-scores do indicate a large increase not only compared to the previous measurement but also compared to the reference population matched for age and gender.

**Fig. 2:**
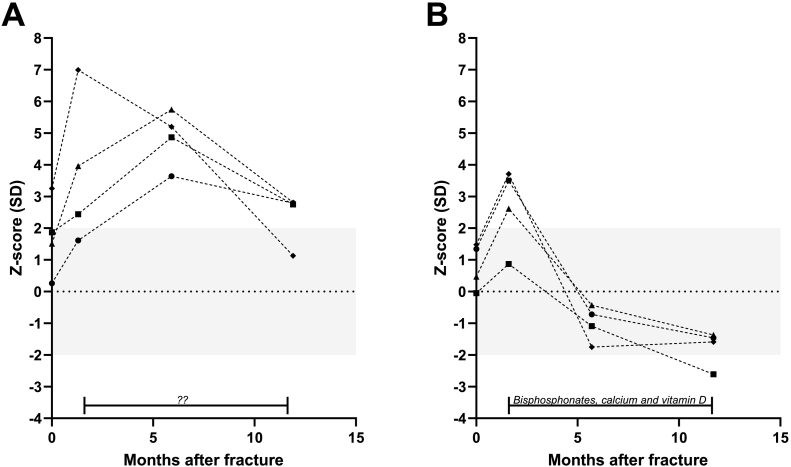
Course at individual patient level of serum levels of sCTX, (♦) OC (■), PINP (▲) and BALP (●) after occurrence of a wrist fracture due to osteoporosis in a 80-year old female who refused to start bisphosphonate treatment (A) and occurrence of a wrist fracture due to osteoporosis in a 66 year old female who did start bisphosphonate, calcium and vitamin D treatment as indicated by the bar and which was continued throughout tsshe depicted follow-up (B).

In Fig. 2, a clear example of the use of BTMs in osteoporosis and the effect of bisphosphonates is depicted. First, there is an increase in bone turnover at the site of the fracture (panel A and B), followed by a rapid decrease in BTM *Z*-scores after the start of bisphosphonate, calcium and vitamin D treatment (panel B), whereas BTM *Z*-scores remain long-term elevated in the patient refusing bisphosphonate treatment (panel A).

## 4 Discussion

This study is the first that describes BTM reference values in a Dutch, Caucasian, population without bone-related abnormalities over the large age range for both female and male individuals. Highest serum levels of BTM were found in childhood and puberty. In adults, BTM levels decrease before reaching stable nadir levels.

We established BTM reference values (of non-fasting blood samples to mimic clinical practice) using 10-year reference cohorts in adults. With our data, *Z*-scores of sCTX, OC, PINP and BALP can be calculated.

The added value of BTM Z-scores using the healthy reference groups described here has been shown in our previous research. sCTX Z-score was included in the MastFx score to identify individual patients with indolent systemic mastocytosis at risk of new fragility fractures (Creighton et al., 1985).

In our prospective cohort study evaluating the short-term and long-term effects of TNF-α blocking therapy on bone metabolism in patients with axial spondyloarthritis, we were able to demonstrate changes over time in all four BTM *Z*-scores (Arends et al., 2012; Siderius et al., 2023). Furthermore, early change in sCTX Z-score was found to be predictive for treatment continuation (Arends et al., 2012). Moreover, baseline levels of sCTX were associated with disease progression defined as formation of syndemophytes after 4 years of TNF-α blocking therapy (Rademacher et al., 2022). In all these studies, both males and females with a large variety in age were included and the use of this healthy reference population made it possible to correct for the normal influence of age and gender by calculating BTM *Z*-scores.

Z-scores enable to compare and combine different studies in a meta-analysis. Furthermore, Z-scores facilitate interpretation at group level and at individual patient level. Whilst it is possible to assess and compare the levels of BTM with regards to the previous measurement during short-term follow-up, longer follow-up and dependent on age or introduction of a therapeutic or disease does alter levels and disallows for comparison. The use of Z-scores allows for a proper comparison independent of time between measurements.

Commercially available assays majorly contributed to the accessibility and usability of BTM, however, there are some important remarks on the data known thus far. Although the kit insert supplied with commercial assays states that each laboratory should establish its own reference values, for practical reasons laboratories often use the manufacturer supplied reference values. These, however, often are limited to certain age groups and do not contain data on the selected reference population making it unclear whether the data represents the population and their BTM levels in the geographical location of the laboratory. BTM activity peaked during childhood and puberty, but also vary with age in adults. With regards to published studies describing BTM levels, most studies did not assess the course and absolute values of BTM in adults, limiting their usability.

Previous studies demonstrated that BTM are clinically relevant biomarkers reflecting the process of bone metabolism, which is eventually related to bone-related outcome. However, in general, there are some important side notes when interpreting BTM data. Numerous biological variables and pre-analytical errors are known to impact serum levels of BTM. First and foremost, the alteration of BTM levels caused by fractures, (bone) diseases (e.g. Paget's and axial SpA) and certain types of medication (e.g. bisphosphonates and hormones) (Ivaska et al., 2007; Coiffier et al., 2013; Eekman et al., 2011; Delmas et al., 2000). Also diseases or physiological changes who do not directly seem related to bone metabolism such as renal insufficiency can impact levels of PINP and OC (Woitge et al., 1999). By nature, levels of BTM will also vary with age, gender and ethnicity (Delmas et al., 2000). Lifestyle factors or habits such as smoking and alcohol usage negatively impact levels of BTM (Marrone et al., 2012; Jorde et al., 2019; Al-Bashaireh and Alqudah, 2020). Movement and weight-bearing exercises are proven to have an osteogenic effect (Creighton et al., 1985; Lombardi et al., 2012). Furthermore, it is known that contraceptives lower BTM levels (Herrmann and Seibel, 2010). Whilst the impact of variation from for example circadian or seasonal variation can be limited using fixed time points for blood withdrawal during longitudinal follow-up of individual patients, other variables are less or uncontrollable (Delmas et al., 2000; Szulc et al., 2017). Additional important aspect is ex vivo alteration of levels caused by pre-analytical errors such as incorrect sampling, delays in blood processing or incorrect storage (Lombardi et al., 2012; Christensen et al., 2019).

Strong aspects of our study are the large and well-selected reference population excluding factors with major influence on BTM as well as the use of common commercially automated assays for the measurement of BTM as it enhances the usability of our reference values. Comparison of our cohort data with the manufacturer supplied reference values does indicate that median and 5-95th percentile BTM levels appear to be slightly higher in comparison to our values in a Western population. For PINP it appears that our healthy reference females aged above 55 years showed lower levels than reference values of manufactures which are population based and can include osteoporotic persons.

There is an ongoing discussion with regards to the change of the assay from Orion RIA to the Roche Elecsys. Whilst the Orion RIA measures intact trimeric PINP, Roche Elecsys measures total PINP. Thus far it appears that in normal healthy individuals there is little difference between measurements. However, in patients with severe renal function impairment, due to lower clearance of degraded monomers of PINP, measurements of total PINP could be higher (Koivula et al., 2012; Koivula et al., 2010; Bhattoa et al., 2021). Therefore, measurement of intact trimeric PINP is a better measure of bone turnover in these patients.

A potential limitation is that information on intoxications (e.g. smoking habit and alcohol intake), movement and weight-bearing exercises and contraceptive pill intake by females was lacking. The reference values for women in the age range 45 to 55 have been derived for premenopausal and postmenopausal women jointly. Despite absence of these data and the possibility that some factors might introduce additional variability with regards to the precision of the results, they are still useful as they do mimic and represent daily clinical practice. For the clinical implementation of our results, it is important to emphasize that our reference population was Caucasian. We decided to collect samples at variable time points during the day from non-fasting individuals in order to reflect the situation of BTM measurement in daily clinical practice. This resulted in a mean “normal” value that is the average circadian CTX level, which is lower compared to previous studies using fasting samples in the morning, and a SD that is somewhat larger due to this variation during the day (Jenkins et al., 2013). For longitudinal follow-up and monitoring of treatment response within individual patients, it is recommended to standardize the collection of blood samples at fixed time points. Furthermore, we used healthy volunteers (i.e. excluding diseases or medication use affecting the bone metabolism as well as osteoporosis after 50 years of age), which results in lower BTM levels compared to population-based studies.

Another limitation of our study is that not all measurements could be performed using one specific lot number of reagents or on one day. However, all BTM measurements were performed in a ISO-certified laboratory. Furthermore, all steps, e.g. lot numbers of reagents and plate readers, were always checked according to the proper local, national and international guidelines and accompanying quality control.

Although the provided reference values for calculating *Z*-scores of BTM already showed interesting results in our previous research, it should be considered as work in progress. Mean and median course of BTM demonstrate good overlap indicating that the distribution within selected age groups was (close to) normal. However, future studies should take into account equality of samples included at certain times of the day, as well as the other limitations that arose in this study.

To conclude, levels of BTM are as expected age and gender dependent. In this study, we provide mean and SD of BTM, which can be used to calculate Z-scores, in reference categories (for age and gender) of Caucasian healthy volunteers. Clinicians can use BTM Z-scores to determine whether there are changes in bone turnover physiology beyond those expected during aging. BTM Z-scores facilitate harmonization of data interpretation and data comparison in daily clinical practice and research settings.

## Abbreviations

BALP: bone-specific alkaline phosphatase
BMD: bone mineral density
BTM: bone turnover marker
DEXA: dual-energy X-ray absorptiometry
OC: osteocalcin
PINP: procollagen type I N-terminal peptide
sCTX: serum C-telopeptide cross-link of type I collagen
SD: standard deviation
SpA: spondyloarthritis
TNF-α: tumor necrosis factor-α
UMCG: university medical center Groningen

## CRediT authorship contribution statement

**Mark Siderius:** Writing – original draft, Formal analysis. **Suzanne Arends:** Writing – review & editing, Formal analysis. **Anneke Muller Kobold:** Writing – review & editing, Investigation. **Lucie Wagenmakers:** Writing – review & editing, Investigation. **Karin Koerts:** Writing – review & editing, Investigation. **Anneke Spoorenberg:** Writing – review & editing, Formal analysis. **Eveline van der Veer:** Writing – review & editing, Investigation, Formal analysis, Conceptualization.

## Declaration of competing interest

The authors declare the following financial interests/personal relationships which may be considered as potential competing interests: Anneke Spoorenberg reports a relationship with 10.13039/100006483AbbVie Inc. that includes: consulting or advisory and funding grants. Anneke Spoorenberg reports a relationship with 10.13039/100004319Pfizer Inc. that includes: consulting or advisory and funding grants. Anneke Spoorenberg reports a relationship with 10.13039/100011110UCB that includes: consulting or advisory and funding grants. Anneke Spoorenberg reports a relationship with MSD that includes: consulting or advisory. Anneke Spoorenberg reports a relationship with Novartis that includes: consulting or advisory.

## Data Availability

Data will be made available on request.
